# The Synergistic Interplay between Vitamins D and K for Bone and Cardiovascular Health: A Narrative Review

**DOI:** 10.1155/2017/7454376

**Published:** 2017-09-12

**Authors:** Adriana J. van Ballegooijen, Stefan Pilz, Andreas Tomaschitz, Martin R. Grübler, Nicolas Verheyen

**Affiliations:** ^1^Department of Health Sciences, Vrije Universiteit Amsterdam and the Amsterdam Public Health Research Institute, Amsterdam, Netherlands; ^2^Division of Endocrinology and Diabetology, Department of Internal Medicine, Medical University of Graz, Graz, Austria; ^3^Department of Epidemiology and Biostatistics, VU University Medical Center and the Amsterdam Public Health Research Institute, Amsterdam, Netherlands; ^4^Bad Gleichenberg Clinic, Bad Gleichenberg, Austria; ^5^Department of Cardiology, Swiss Cardiovascular Center Bern, Bern University Hospital, University of Bern, Bern, Switzerland; ^6^Department of Cardiology, Medical University of Graz, Graz, Austria

## Abstract

Vitamins D and K are both fat-soluble vitamins and play a central role in calcium metabolism. Vitamin D promotes the production of vitamin K-dependent proteins, which require vitamin K for carboxylation in order to function properly. The purpose of this review is to summarize available evidence of the synergistic interplay between vitamins D and K on bone and cardiovascular health. Animal and human studies suggest that optimal concentrations of both vitamin D and vitamin K are beneficial for bone and cardiovascular health as supported by genetic, molecular, cellular, and human studies. Most clinical trials studied vitamin D and K supplementation with bone health in postmenopausal women. Few intervention trials studied vitamin D and K supplementation with cardiovascular-related outcomes. These limited studies indicate that joint supplementation might be beneficial for cardiovascular health. Current evidence supports the notion that joint supplementation of vitamins D and K might be more effective than the consumption of either alone for bone and cardiovascular health. As more is discovered about the powerful combination of vitamins D and K, it gives a renewed reason to eat a healthy diet including a variety of foods such as vegetables and fermented dairy for bone and cardiovascular health.

## 1. Introduction

Worldwide, a large group of people is prescribed to a supplemental regime of both vitamin D and calcium. In Europe, depending on a country and sex, between 1 and 66% of the adult population use vitamin D supplements [1, 2]. Over the last decade, large vitamin D supplementation is promoted to restore 25-hydroxyvitamin D (25(OH)D) concentrations and is considered to be safe with doses up to 4000 international units (IU) per day [3]. However, little is known about potential long-term high-dose vitamin D supplementation [2, 4].

Vitamin D is a fat-soluble vitamin that can be ingested by foods such as fatty fish, dairy products, and eggs, but is mainly synthesized by the human skin when exposed to sunlight. In the liver, vitamin D is hydroxylated to 25(OH)D, the main circulating vitamin D metabolite that is measured to assess and classify vitamin D status. Circulating 25(OH)D is further metabolized by the kidney for full biological activity into its most active form 1,25-dihydroxyvitamin D (1,25(OH)D) also known as calcitriol. Calcitriol is also produced endogenously by extrarenal production through peripheral 1-*α*-hydroxylase and has positive effects on immune function and anticancer activity [5–7]. Vitamin D plays a main role in regulating calcium metabolism by increasing intestinal calcium absorption [8]. Ample evidence recommends vitamin D supplementation for the prevention of falls and fractures [9, 10]; however, evidence suggests calcium precipitation in the vasculature and other potential side effects [4, 11–14].

Vitamin K is another fat-soluble vitamin that exists in two forms of vitamin K: vitamin K_1_ (phylloquinone, mainly found in green leafy vegetables) and vitamin K_2_ (menaquinone, mainly found in fermented dairy and produced by lactic acid bacteria in the intestine) [15]. Vitamin K stores are limited, but they can be recycled to a certain extent [16]. Vitamin K_1_ is principally transported to the liver, regulating the production of coagulation factors, while vitamin K_2_ is transported to extrahepatic tissues, such as bone and the vascular wall, regulating the activity of matrix Gla protein (MGP) and osteocalcin (bone Gla protein)—the main vitamin K-dependent proteins. They require vitamin K for carboxylation in order to function properly. When circulating concentrations of vitamin K are insufficient, a greater proportion of MGP and osteocalcin remain uncarboxylated, which is associated with unfavorable outcomes such as cardiovascular disease, lower BMD, and osteoporosis [17]. The current recommendation for vitamin K_1_ intake is 70 *μ*g/day for all adults defined by an adequate intake [18]. This amount is solely based on maintaining coagulation function and might not be enough for optimal carboxylation of other vitamin K-dependent proteins, which require higher amounts of vitamin K [19].

The role of vitamin K in cardiovascular health has mainly been studied in isolation [20]; however, a growing body of evidence suggests a synergistic effect of vitamin K combined with vitamin D [21–26]. Vitamin D promotes the production of vitamin K-dependent proteins, as shown in rats by Karl et al. already in 1985 [27]. These findings cannot be explained by our current understanding of the biochemical role of vitamin K, but suggest that vitamin D may influence vitamin K-dependent proteins [28].

The purpose of this narrative review is to summarize available evidence in the field of the synergistic interplay between vitamins D and K on bone and cardiovascular health. The primary focus is on the general population and includes observational studies that investigated both vitamin D and vitamin K status with outcome measures and supplementation studies that administered both vitamins D and K.

## 2. Interaction of Vitamins D and K for Bone Health

### 2.1. Experimental Studies

In experimental models, the exploration of the interaction between vitamins D and K on bone health is ongoing for decades and a fair amount of literature is available. Recent understanding suggests that vitamin D enhances vitamin K-dependent bone protein concentrations and induces bone formation in vitro [29–31] with stimulation of osteoblast-specific gene expression [32]. Osteoblast-specific expression of osteocalcin is controlled at the transcriptional level by 1,25(OH)D through the 1,25(OH)D-responsive element within the promoter of the osteocalcin gene [32]. The underlying mechanism of mineralization induced by vitamin K in the presence of 1,25(OH)D was different from vitamin K alone [33]. In rats, 1,25(OH)D receptor binding can undergo gamma-carboxylation in the presence of vitamin K. This means that 1,25(OH)D receptor carboxylation can potentially modify the intrinsic biochemical properties of the nuclear receptors and modulates its binding to DNA [34].

The effect of 1,25(OH)D and warfarin—a vitamin K antagonist—on the vitamin K cycle was studied in cultured osteoblasts [26]. Epoxide reductase, one of the key enzymes in the vitamin K cycle, was strongly inhibited by warfarin, whereas it was not affected by 1,25(OH)D, meaning that the vitamin K metabolic cycle functions normally in human osteoblasts.

Human osteoblast cell cultures indicate that glycoxidation interferes with the maturation of osteoblasts; however, this process may be counterbalanced by adding vitamins D and K, which reverses the detrimental glycoxidation on several bone markers [35]. Therefore, the addition of vitamins D and K may induce important biochemical changes in bone, which may exert therapeutic effects on bone metabolic diseases such as osteoporosis [36].

### 2.2. Animal Models

A growing body of evidence is also documenting the interaction between vitamins D and K in animal models. The effect of vitamin K of bone mineralization is enhanced by plasma 25(OH)D concentration. Vitamin K was administered to prevent osteoporosis in ovariectomized rats, but bone loss was only prevented in rats fed with a diet containing vitamin D or vitamin D supplementation [37, 38]. These findings suggest that combined treatment with vitamins D and K is more effective than vitamin K alone particularly in the early phase of estrogen deficiency after menopause.

Vitamin K and vitamin D supplementation on calcium balance was investigated in young rats fed with a normal or low calcium diet, plus vitamin K and/or vitamin D [39]. Vitamin K supplementation promoted the reduction in urinary calcium excretion and stimulated intestinal calcium absorption in rats on a normal calcium diet. Vitamin D supplementation stimulated intestinal calcium absorption with prevention of the abnormal elevation of serum PTH concentrations, prevented hypocalcemia in rats fed with a low calcium diet, and stimulated intestinal calcium absorption in rats fed with a normal calcium diet. The stimulation of intestinal calcium absorption was associated with increased 1,25(OH)D concentrations. An additive effect of vitamin K and vitamin D on intestinal calcium absorption was only found in rats fed with a normal calcium diet. This study shows the differential effects of vitamin K and vitamin D supplementation on calcium balance in young rats fed with a normal or low calcium diet.

### 2.3. Observational Evidence

Human evidence for the role of 1,25(OH)D in stimulating vitamin K-dependent proteins is scarce. In hemodialysis patients, vitamin D analog users had much higher concentrations of bone Gla protein (BGP) than nonusers indicating that vitamin D administration may play a role in stimulating vitamin K-dependent protein activity [40]. More research on the stimulating role of vitamin D on vitamin K-dependent proteins is urgently needed to study the underlying mechanisms.

Some observational studies support the hypothesis that optimal concentrations of both vitamins D and K support bone mineralization and lower fracture risk. In a cross-sectional study among Japanese older men, lower 25(OH)D and vitamin K_1_ concentrations were concomitantly associated with BMD, indicating a nonestrogen-dependent pathway in men [41]. In a case-control study of 184 Norwegian older adults, the combination of low vitamin K_1_ and low 25(OH)D was synergistically associated with hip fractures: odds ratio 7.6 (95% CI 2.3, 26.7) [42]. In the NOREPOS study, another Norwegian population study, similar results were observed among 1318 older adults [43]. During 8.2-year follow-up, the combination of both low vitamin D and K_1_ concentrations was associated with a greater hip fracture risk, hazard ratio 1.41 (95% CI 1.09, 1.82), compared to the high vitamin D and vitamin K category. No increased risk was observed in the groups low in 1 vitamin only. These results indicate that the combination of low concentrations of vitamin K_1_ and 25(OH)D is associated with increased risk of hip fractures.

### 2.4. Human Intervention Studies

A small study among 15 healthy women indicated that 3 weeks of supplementation of 20 ml extra virgin olive oil enriched with vitamins D, K, and B_6_ resulted in lower concentrations of uncarboxylated osteocalcin [44]. This means that a vitaminized oil can influence vitamin K-dependent proteins within multiple weeks.

An increasing amount of randomized controlled trials have demonstrated the combined effects of vitamins D and K on postmenopausal osteoporosis mostly pursued in Japan with a study duration between 8 weeks and 3 years (Table 1). A randomized trial with 4 arms (diet, menaquinone-4, cholecalciferol, and menaquinone-4 + cholecalciferol) showed that only the vitamin K plus vitamin D arm increased BMD [45]. Similar results were found in another trial with postmenopausal women with osteoporosis ≥ 5 years after menopause [46]. After 2 years of follow-up, the longitudinal changes in BMD were significant compared with those in the calcium lactate-, vitamin D-, and vitamin K-only groups (*P* < 0.001). A modest synergistic effect of vitamins D and K was found after 2 years in healthy older women from nutritionally relevant intakes of vitamin K_1_ together with supplements of calcium plus vitamin D_3_ on bone mineral concentration compared to either vitamin D or K alone or placebo [47]. The complementary effect of vitamin K_1_ (1 mg/day) and a mineral + vitamin D supplement (8 *μ*g/day) was most effective in reducing bone loss at the femoral neck after 3 years among postmenopausal women versus vitamin D alone or placebo [48]. The addition of vitamin K to vitamin D and calcium supplements compared to vitamin D and calcium alone in postmenopausal Korean women increased BMD and reduced uncarboxylated osteocalcin concentrations after 6 months compared to vitamin D and calcium alone [49]. In postmenopausal women, 1 year of oral supplementation with extra virgin olive oil enriched with vitamins D_3_, K_1_, and B_6_ or extra virgin olive oil reduced uncarboxylated osteocalcin concentrations and increased the T-score of BMD [50]. These findings indicate that combined administration of vitamin D and vitamin K appears to be useful in increasing BMD in postmenopausal women. It should be noted that these studies found beneficial effects at some but not all BMD sites measured. Furthermore, treatment with vitamins D and K with calcium increased BMD in older female patients with Alzheimer's disease and led to the prevention of nonvertebral fracture odds ratio: 7.5 (95% CI 5.6, 10.1); however, no placebo capsules were administered, hampering the interpretation of the results [51].

Not all studies observed synergistic effects of vitamin D and K supplementation. A small study among adults with Crohn's disease in Ireland showed generally no effect of combined vitamin D and K supplementation versus placebo on bone mass after 1 year, except a modest increase in bone mass of the total radius [52]. Among healthy women, 1 year of vitamin D and calcium + vitamin K supplementation either by phylloquinone or menaquinone-4 supplementation had no effect on BMD compared to calcium and vitamin D alone [53]. This study does not support a combined role for vitamin D + K supplementation in osteoporosis prevention; however, the relatively short study duration and the inclusion of healthy women could explain the null finding. It is however questionable if BMD can be improved in 12 months since changes in BMD usually require at least 1 year of follow-up time.

Among healthy older men and women, no difference was observed between multivitamin and calcium and vitamin D compared with the addition of vitamin K on BMD after 3 years [19]. An additive effect was noticeable for decreased percentage of uncarboxylated osteocalcin, which indicates an improved vitamin K status in the treatment group.

The ECKO trial among postmenopausal women with osteopenia showed no beneficial effect of vitamin D and calcium + vitamin K supplementation versus vitamin D and calcium alone after 2 years of follow-up in vitamin D-sufficient women [54]. However, the risk of fractures—a clinically more meaningful endpoint—was lower in the vitamin D and calcium + vitamin K groups: hazard ratio 0.41 (95 CI 0.1, 1.18) at 2 years and 0.45 (95% CI 0.20, 0.98) after 4 years of follow-up. This result on fracture risk indicates that bone quality rather than quantity is more important as not all trials showed synergistic effects of vitamin D and K supplementation on bone mineral density.

The protective effect of vitamin D with K on prednisolone-induced loss of BMD in patients with chronic glomerulonephritis after 8 weeks of treatment was similar in the vitamin D-only group [55], meaning that the addition of vitamin K had no synergistic effect. The elevation in serum calcium concentrations in the vitamin D group was, however, attenuated in the vitamin D + K group.

Taken together the evidence for combined vitamin D and K supplementation, the majority of the studies found beneficial effects for BMD among postmenopausal women.

## 3. Interaction between Vitamins D and K for Cardiovascular Health

Besides bone health, also, the interaction between vitamins D and K with regard to cardiovascular health receives growing research interest. MGP—the vascular marker of vitamin K status—needs *γ*-glutamate carboxylation to inhibit vascular calcification [56]. In an experimental rat model, warfarin was administered to induce vitamin K deficiency and caused arterial calcification [57], which was accelerated when given toxic doses of vitamin D and resulted in premature death.

The Czech MONICA study cross-sectionally observed that subjects in the highest quartile of dephosphorylated-uncarboxylated MGP (dp-ucMGP) plus the lowest quartile of 25(OH)D concentrations had the highest pulse wave velocity in middle-aged healthy adults [58]. Further, potential interaction between vitamin K status and polymorphisms of the vitamin D receptors was investigated. Pulse wave velocity was higher with the number of G-allele polymorphisms and highest in the top quartile of dp-ucMGP for the GG vitamin D receptor genotype.

A Dutch prospective cohort indicates that the combination of low vitamin D < 50 mmol/L and low K status ≥ 323 mmol/L dp-ucMGP was associated with increased systolic and diastolic blood pressures and incident hypertension after 6 years of follow-up [59]. Up to now, no study investigated the combination of optimal vitamin D and K status in relation to coronary artery calcification and cardiovascular events after long-term follow-up. This would give valuable insight if vitamins D and K are involved in developing cardiovascular disease.

So far, two human intervention studies in healthy populations have investigated the combined effect of vitamins D and K on vascular function and calcification (Table 2) [60, 61]. In postmenopausal women, after 3 years of supplementation (1000 *μ*g/d vitamin K_1_ + 320 IU vitamin D), the vitamin D + K group maintained vessel wall characteristics of the carotid artery, whereas the control group and the vitamin D-only group significantly worsened over 3 years of follow-up [60]. However, vitamin K status was not measured as a marker of compliance to investigate what would have occurred following supplementation. Further, in a 3-year, double-blind, randomized controlled trial in older men and women free of clinical CVD, daily supplemental vitamin K in amounts achievable by high dietary intake of green, leafy vegetables (500 *μ*g/day) combined with 600 mg calcium carbonate and 10 *μ*g (400 IU) vitamin D did not result in lower coronary artery calcium progression as assessed by computerized tomography compared to the calcium + vitamin D group. In a subgroup analysis of participants who were ≥85% adherent to supplementation, there was less coronary artery calcium progression in the vitamin K + calcium and vitamin D groups than in the calcium and vitamin D group alone [61]; however, MGP carboxylation status was not determined. These data are hypothesis generating, and further studies are warranted to clarify the mechanism.

Among overweight type 2 diabetic patients with coronary heart disease, cosupplementation for 12 weeks of vitamins D (10 *μ*g) and K (180 *μ*g) and calcium (1000 mg) had beneficial effects on maximum levels of left carotid intima-media thickness and insulin metabolism markers [62]; however, no effect on right intima-media thickness was found and the results could be a chance finding. Unfortunately, circulating markers of vitamin K concentrations and vitamin K-dependent proteins were not taken into account to get a better mechanistic understanding.

Two trials studied the effect of vitamin D versus vitamin D + K in nondialyzed CKD patients on vascular calcification and cardiovascular risk factors for 9 months [63, 64]. In 42 CKD patients, the increase in carotid intima-media thickness (IMT) was significantly lower in the K (90 *μ*g menaquinone-7) + D (10 *μ*g vitamin D) group compared with the D-only group after 9 months [63]. Another small trial (*n* = 38) from the same research group did not show differences between the D versus D + K groups on cardiovascular risk markers [64]. These few studies show some potential for the combined effect of vitamins D + K versus D alone on subclinical CVD risk markers. It should be noted that very few clinical studies have been conducted in this field and that vitamin D + K supplements have been often combined with different micronutrients making it difficult to solely pinpoint the effect of vitamin D + K. These limited studies indicate that joint supplementation might benefit cardiovascular health.

## 4. Vitamins D and K with Glucose Metabolism and Inflammation

Another pathway that might affect CVD risk is via disturbances in glucose metabolism. Among Iranian vitamin D-deficient women with polycystic ovary syndrome—a dysmetabolic disorder—cosupplementation of calcium (1000 mg) and vitamins D (400 IU) and K (180 *μ*g) for 8 weeks improved markers of insulin metabolism and lipid concentrations compared to placebo [65]. The joint supplementation of vitamins D and K might improve insulin metabolism through an effect on upregulation of the insulin receptor genes, the regulation of insulin secretion from the pancreatic beta-cell, the enhancement of *β*-cell proliferation, and suppression of parathyroid hormone [66–69].

Further, another feature in which both vitamins D and K overlap is on inflammation, which is strongly related to the development of CVD and osteoporosis [70]. In the same Iranian clinical trial among vitamin D-deficient women with polycystic ovary syndrome, the joint supplementation of calcium with vitamins D and K had beneficial effects on endocrine and oxidative stress markers, however no effect on inflammatory markers [71].

## 5. Effects of Long-Term Vitamin D Supplementation

**Figure 1:**
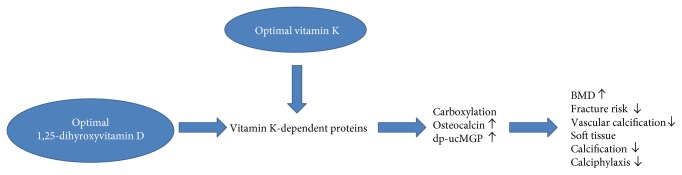
Simplified overview of potential synergy between vitamins D and K and bone and cardiovascular health. dp-ucMGP: dephosphorylated-uncarboxylated matrix Gla protein: BMD: bone mineral density. Genetic, molecular, cellular, and human evidence support that optimal concentrations of both vitamin D and vitamin K are beneficial for bone and cardiovascular health. Vitamin K is needed for the carboxylation of vitamin K-dependent proteins such as osteocalcin and matrix Gla protein, while vitamin D promotes the production of vitamin K-dependent protein concentrations. These vitamin K-dependent proteins are needed for extrahepatic organs such as the bone and the vascular system. This will result in bone mineralization and will inhibit soft tissue calcification, which will ultimately lead to lower risks of fractures and coronary heart disease.

A large group of people uses both vitamin D and calcium for the prevention of falls and fractures. Given the fact that 25(OH)D is converted to 1,25(OH)D, vitamin D supplementation stimulates the production of 1,25(OH)D [72]. This means that long-term vitamin D supplementation could promote the production of large amounts of vitamin K-dependent proteins, which remain inactive because there is not enough vitamin K to carboxylate (Figure 1()). We propose a new hypothesis that if vitamin D concentrations are constantly high, there might not be enough vitamin K for activation of vitamin K-dependent proteins. Consequently, excess vitamin D diminishes the ability of vitamin K-dependent proteins to function properly, to stimulate bone mineralization, and to inhibit soft tissue calcification.

Further, increasing vitamin D intake through dietary or supplemental source increases intestinal calcium absorption, particularly when combined with calcium supplementation, and promotes hypercalcemia [73]. In this context, a human trial was performed in older women who received either 1200 mg calcium or 1200 mg calcium and 800 IU vitamin D per day over a 12-week period [74]. At the end of the 12 weeks, neither group observed a change in calcium concentrations, meaning that calcium was either excreted or stored somewhere. Increased calcium intake by itself may not be problematic as long as there is a steady state between optimal vitamin D and vitamin K concentrations. The disbalance between vitamin D and vitamin K promotes an environment in which excess calcium will be deposited into our vascular tissue instead of bone. The migration of calcification into the vascular tissue is described by the double burden of atherosclerosis and osteoporosis [75–77]. Additionally, as vitamin D increases calcium absorption, it might also promote hypercalcemia as seen in the Women's Health Initiative, which found a 24% higher risk of myocardial infarction in individuals taking calcium and vitamin D supplements and a greater risk for urinary tract stone occurrence: hazard ratio 1.17 (95% CI 1.02, 1.34) [11, 13, 14]. One prospective study found that higher 1,25(OH)D concentrations were strongly associated with the incidence of hypertension, while 25(OH)D was inversely associated with hypertension risk [78]. Higher 1,25(OH)D was associated with lower urinary calcium excretion, which could mean that the calcium meant for bone is stored somewhere else. Unfortunately, vitamin K status was not measured which would have given valuable insight into the association between vitamins D and K with calcium excretion.

## 6. Calciphylaxis and Vitamin K Antagonist Use

Calciphylaxis is a syndrome of calcification of the blood vessels, coagulopathy, and skin necrosis. It is seen mostly in patients with end-stage kidney disease, but can occur in the absence of kidney failure. Vitamin K antagonist use may contribute to its development [79]. The syndrome may cause a substantial morbidity and mortality. However, it should be acknowledged that the term calciphylaxis refers to a heterogeneous disorder that is characterized by soft tissue and vascular necrosis and has a clinical presentation from mild to severe. The underlying causes of calciphylaxis are not well understood; however, reported risk factors include female sex, obesity, elevated calcium^∗^phosphate product, warfarin use, and vitamin D derivatives, for example, calcitriol, calcium-based binders, or systemic steroids, low blood albumin concentrations, and type 2 diabetes [80]. A recent study among patients with hemodialysis with calciphylaxis versus hemodialysis showed that cases had higher plasma uncarboxylated MGP concentrations than controls, which suggest a role of MGP in the pathophysiology of calciphylaxis. The fraction of total MGP that was carboxylated was also lower in cases than in controls. Vitamin K deficiency-mediated reduction in relative carboxylated MGP concentration may play a role in the pathogenesis of calciphylaxis [81]. This could be further mediated by the combined use of vitamin D derivatives and warfarin. Further, another study indicated that vitamin K antagonist use predisposes to the development of calciphylaxis in end-stage renal disease [82]. More evidence on the combined role of vitamin K antagonist use and vitamin D on bone and cardiovascular health is urgently needed.

## 7. Vitamin D and K Supplementation

Based on the current body of evidence, there is not enough evidence to recommend combined vitamin D and K supplementation for the prevention and treatment of osteoporosis. Most trials studied low-dose vitamin D in isolation (400–800 IU daily), which demonstrated only modest or null effects on BMD and fracture prevention in mostly ≥65 years postmenopausal women [6–8]. Large clinical trials of moderate–high dose (≥800 IU daily) vitamin D supplementation (cholecalciferol) are currently in progress.

The most widely used vitamin K form for supplementation is vitamin K_2_ and more specifically menaquinone-4 and menaquinone-7. Menaquinone-4 is more used in trials with bone outcomes, while menaquinone-7 is more in trials with cardiovascular outcomes with dosages between 90–360 *μ*g. Menaquinone-7 has a higher bioavailability and may be of particular importance for extrahepatic tissue [83]. No cut-off value for vitamin K status nor vitamin K supplementation is available yet. Future studies are needed to determine whether vitamin D combined with vitamin K rich foods or vitamin K supplementation could improve bone and cardiovascular health.

## 8. Recommendations for Future Research

The recommendations for future research are as follows:

- Evaluate the role of vitamin D administration in vitamin K-dependent proteins in human populations
- Question the possible long-term consequences of high-dose vitamin D supplementation
- Assess the combined role of vitamin K antagonist use and vitamin D in bone and cardiovascular health
- Investigate the joint supplementation of vitamins D and K on hard clinical endpoints

## 9. Conclusion

Taken together, animal and human studies suggest that optimal concentrations of both vitamin D and vitamin K are beneficial for bone and cardiovascular health as supported by genetic, molecular, cellular, and some human studies. However, vitamin D and calcium supplementation along with vitamin K deficiency might also induce long-term soft tissue calcification and CVD, particularly in vitamin K antagonist users and other high-risk populations. At this moment, we should be careful about supplementing high-dose vitamin D, unless indicated differently. More clinical data about the potential interplay between vitamin D and vitamin K metabolism is urgently needed before broader treatment recommendations can be given.

The consumption of a well-balanced diet is key for population-based primary prevention of chronic diseases. As more is discovered about the powerful combination of vitamins D and K, it gives a renewed reason to eat a healthy diet including a variety of foods such as vegetables and fermented dairy for bone and cardiovascular health.

## Figures and Tables

**Table 1:** Summary of clinical trials of combined vitamin D and K supplementation on bone health.

Author, year	Country	Participants	Treatment	Study duration	Outcome	Results for the highest versus the lowest quartiles
Iwamoto et al., 2000 [46]	Japan	*N* = 92 osteoporotic women ≥ 5 years after menopause, mean age 64 years	(i) Calcium (calcium lactate, 2 g/day) (ii) Vitamin D_3_ 0.75 *μ*g/day (iii) Vitamin K_2_ 45 mg/day (iv) Vitamin D_3_ plus vitamin K_2_	2 years	Bone mineral density % change	Combined vitamins D and K increased BMD
Ushiroyama et al., 2002 [45]	Japan	*N* = 126 postmenopausal women with osteopenia and osteoporosis, mean age 53 years	(i) Diet (ii) Vitamin K_2_ 45 mg/day MK-4 (iii) 1-*α* hydroxylcholecalciferol 1 *μ*g/day (iv) Vitamin K_2_ + 1-*α* hydroxylcholecalciferol	2 years	Bone mineral density % change	K + D group increased BMD % change at 2 years *P* < 0.001
Braam et al., 2003 [48]	Netherlands	*N* = 155 postmenopausal women between 50 and 60 years	(i) Placebo (ii) Mineral + vitamin D (8 *μ*g/day) (iii) Mineral + vitamin D + vitamin K_1_ 1 mg	3 years	Bone loss	Mineral + vitamin D + vitamin K showed reduced bone loss of the femoral neck
Yonemura et al., 2004 [55]	Japan	*N* = 60 patients with chronic glomerulonephritis, mean age 32 years, 53% female	(i) Control (ii) Vitamin D (alfacalcidol 0.5 mg) (iii) Vitamin K_2_ MK-4 45 mg/d (iv) Vitamins D plus K	8 weeks	Bone mineral density	The preventive effect in groups K and D + K was similar to D
Sato et al., 2005 [51]	Japan	*N* = 200 older women with Alzheimer's disease, mean age 78 years	(i) Placebo (ii) 45 mg menatetrenone, 1000 IU ergocalciferol, and 600 mg calcium	2 years	Bone mineral density and fractures	BMD increased in vitamin D + K group Odds ratio nonvertebral fractures 7.5 (95% CI 5.6, 10.1)
Bolton-Smith et al., 2007 [47]	UK	*N* = 244 healthy women, mean age 68 years	(i) Placebo (ii) 200 mg/d vitamin K_1_ (iii) 400 IU vitamin D_3_ + 1000 mg calcium (iv) Vitamins K_1_ and D_3_ plus calcium	2 years	Bone mineral content	Combined vitamin K with vitamin D plus calcium associated with an increase in bone mineral content at the ultradistal radius
Booth et al. 2008 [19]	US	*N* = 401 healthy men and women, mean age 69, 59% female	(i) Multivitamin + 10 *μ*g vitamin D and 600 mg calcium (ii) Multivitamin + vitamin D + calcium + 500 *μ*g vitamin K_1_	3 years	Bone mineral density	No differences in change in BMD Vitamin D + K group lower uncarboxylated osteocalcin concentrations
Cheung et al., 2008 [54]	Canada	*N* = 440 postmenopausal women with osteopenia, mean age 59 years	(i) 1500 mg calcium + 800 IU vitamin D (ii) 5 mg of vitamin K_1_ + calcium and vitamin D	2–4 years	Bone mineral density	No effect on BMD
Binkley et al., 2009 [53]	US	*N* = 381 postmenopausal women, mean age 62 years	(i) Calcium 315 mg + vitamin D_3_ 200 IU (ii) Phylloquinone 1 mg + calcium and vitamin D_3_ (iii) MK-4 (45 mg day) + calcium and vitamin D_3_	1 year	Bone mineral density	No effect on BMD
Je et al., 2011 [49]	South Korea	*N* = 78 Korean postmenopausal women, mean age 68 years	(i) Vitamin D 400 IU + calcium (630 mg) (ii) Vitamin D + calcium +45 mg of vitamin K_2_	6 months	Bone mineral density	BMD increased significantly in the vitamin D + K group
O'Connor et al., 2014 [52]	Ireland	*N* = 46 adults with Crohn's disease, mean age 45 years	(i) Placebo (ii) Phylloquinone 1 mg, vitamin D 10 *μ*g, and calcium 500 mg/d	1 year	Bone mineral density	Small effect on BMD of the total radius for vitamin D + K group
Mazzanti et al., 2015 [50]	Italy	60 healthy postmenopausal women, mean age 55 years	(i) Extra virgin olive oil (ii) Extra virgin olive oil enriched with vitamins D_3_, K_1_, and B_6_	1 year	Bone mineral density	Vitaminized oil D, K, and B_6_ increased the T-score of BMD

BMD: bone mineral density; MK-4: menaquinone-4.

**Table 2:** Summary of clinical trials of combined vitamin D and K supplementation on cardiovascular health and disease.

Author, year	Country	Participants	Treatment	Study duration	Outcome	Results for the highest versus the lowest quartiles
Braam et al., 2004 [60]	Netherlands	*N* = 181 postmenopausal women, means age 55, 100% female	(i) Placebo (ii) Minerals + 8 *μ*g vitamin D (iii) Minerals + 8 *μ*g vitamin D + 1 mg vitamin K_1_	3 years	Vessel wall characteristics	MDK group unchanged, placebo and minerals + vitamin D decreased elastic properties
Shea et al., 2009 [61]	US	*N* = 388 healthy men and postmenopausal women, mean age 66 y, 60% female	(i) Multivitamin + 10 *μ*g vitamin D and 600 mg calcium (ii) Multivitamin + vitamin D + calcium + 500 *μ*g vitamin K_1_	3 years	Coronary artery calcification	No difference between vitamin K_1_ group and control group
Asemi et al., 2016 [62]	Iran	*N* = 66 overweight diabetic patients with coronary heart disease, mean age 65 y, 47% female	(i) Placebo (ii) Vitamin D (10 *μ*g), K (180 *μ*g), and calcium (1000 mg)	12 weeks	Carotid IMT	Lower left carotid intima-media thickness and improved insulin metabolism markers
*Chronic kidney disease patients*						
Kurnatowska et al., 2015 [63], 2016 [64]	Poland	*N* = 42 nondialyzed CKD patient stages, mean age 60 y, 3–5, 45% female	(i) 10 *μ*g cholecalciferol (ii) 10 *μ*g cholecalciferol + 90 *μ*g MK-7	270 days	Carotid IMT	Reduced progression IMT, reduced dp-ucMGP and osteocalcin

IMT: intima-media thickness; dp-ucMGP: dephosphorylated-uncarboxylated matrix Gla protein; MK-7: menaquinone-7.
